# Blocking connexin43 hemichannels protects mice against tumour necrosis factor-induced inflammatory shock

**DOI:** 10.1038/s41598-019-52900-4

**Published:** 2019-11-12

**Authors:** Tinneke Delvaeye, Maarten A. J. De Smet, Stijn Verwaerde, Elke Decrock, Aleksandra Czekaj, Roosmarijn E. Vandenbroucke, Kelly Lemeire, Amanda Gonçalves, Wim Declercq, Peter Vandenabeele, Dmitri V. Krysko, Luc Leybaert

**Affiliations:** 1 VIB Center for Inflammation Research, Ghent, Belgium; 2 Department of Biomedical Molecular Biology, Ghent University, Ghent, Belgium; 3 Department of Basic and Applied Medical Sciences, Ghent University, Ghent, Belgium; 4 VIB BioImaging Core, Ghent, Belgium; 5 Methusalem Program, Ghent University, Ghent, Belgium; 6 Department of Human Structure and Repair, Ghent University, Ghent, Belgium

**Keywords:** Membrane proteins, Infectious diseases, Calcium signalling, Cell death

## Abstract

Upon intravenous injection of tumour necrosis factor (TNF) in mice, a systemic inflammatory response syndrome (SIRS) is initiated, characterized by an acute cytokine storm and induction of vascular hyperpermeability. Connexin43 hemichannels have been implicated in various pathological conditions, e.g. ischemia and inflammation, and can lead to detrimental cellular outcomes. Here, we explored whether targeting connexin43 hemichannels could alleviate TNF-induced endothelial barrier dysfunction and lethality in SIRS. Therefore, we verified whether administration of connexin43-targeting-peptides affected survival, body temperature and vascular permeability *in vivo*. *In vitro*, TNF-effects on connexin43 hemichannel function were investigated by single-channel studies and Ca^2+^-imaging. Blocking connexin43 hemichannels with TAT-Gap19 protected mice against TNF-induced mortality, hypothermia and vascular leakage, while enhancing connexin43 hemichannel function with TAT-CT9 provoked opposite sensitizing effects. *In vitro* patch-clamp studies revealed that TNF acutely activated connexin43 hemichannel opening in endothelial cells, which was promoted by CT9, and inhibited by Gap19 and intracellular Ca^2+^-buffering. *In vivo* experiments aimed at buffering intracellular Ca^2+^, and pharmacologically targeting Ca^2+^/calmodulin-dependent protein kinase-II, a known modulator of endothelial barrier integrity, demonstrated their involvement in permeability alterations. Our results demonstrate significant benefits of inhibiting connexin43 hemichannels to counteract TNF-induced SIRS-associated vascular permeability and lethality.

## Introduction

During TNF-induced SIRS, an exaggerated production and secretion of pro-inflammatory cytokines (e.g. interleukin 6 (IL-6), IL-1) and chemokines (CXCL-1) into the circulation results in the activation and increased permeability of the endothelium^1–4^. Endothelial cells (ECs) are connected by different junctional proteins that mediate vascular barrier function and intercellular communication^5^. The reorganization of tight and adherens junctions has been frequently linked to microvascular dysfunction and hyperpermeability, often induced by TNF and other inflammatory mediators^6–8^. However, little is known on the role of connexins (Cxs) and their channels, i.e. gap junction channels (GJCs) and hemichannels, in the pathogenesis of SIRS. GJCs consist of two hemichannels, each containing 6 Cx proteins. The latter are composed of four transmembrane regions, two extracellular loops, a cytoplasmic loop and cytosolic N- and C-terminal tails. Hemichannels provide a passageway between the intra- and extracellular environment, while GJCs connect the cytoplasm of two neighbouring cells, providing a route for direct cell-cell communication. Both hemichannels and GJCs allow the passage of ions and small molecules (≤1.5 kDa) such as adenosine trisphosphate (ATP) and inositol trisphosphate (IP_3_)^9,10^. Cx43 is the most abundant and widespread Cx in mammals, including in vascular ECs^11,12^. Cx43 hemichannels are primarily closed under physiological conditions^13,14^; however, they are reported to open upon various, mostly pathological stimuli such as ischemic or inflammatory conditions^15,16^. Given the large conductance of Cx43 hemichannels, excessive opening of these channels is expected to have deleterious consequences for the integrity of the cell due to dissipative electrical current flow, disturbed cellular ion homeostasis, Ca^2+^-entry and loss of crucial metabolites such as e.g. ATP or glutamate^17,18^.

Our aim was to investigate whether selectively targeting Cx43 hemichannels could rescue animals from TNF-induced SIRS, particularly from vascular leakage. We targeted Cx43 hemichannels *in vivo* by making use of TAT-Gap19^19^ and TAT-CT9^20–22^, respectively known to block or promote Cx43 hemichannel opening (for a review, see^11,23^). Gap19 and CT9 both have their targets inside the cell, at the C-terminal tail and cytoplasmic loop of Cx43, respectively, and are therefore fused to a translocation sequence (TAT) to improve their membrane permeation *in vivo*. Combined, our *in vivo* and *in vitro* approaches demonstrate that Cx43 hemichannels, through TNF-induced Ca^2+^-increases, lead to renal vascular permeability and animal mortality in SIRS.

## Results

### Blocking Cx43 hemichannels protects mice against TNF-induced mortality, hypothermia and vascular permeability alterations

**Figure 1:**
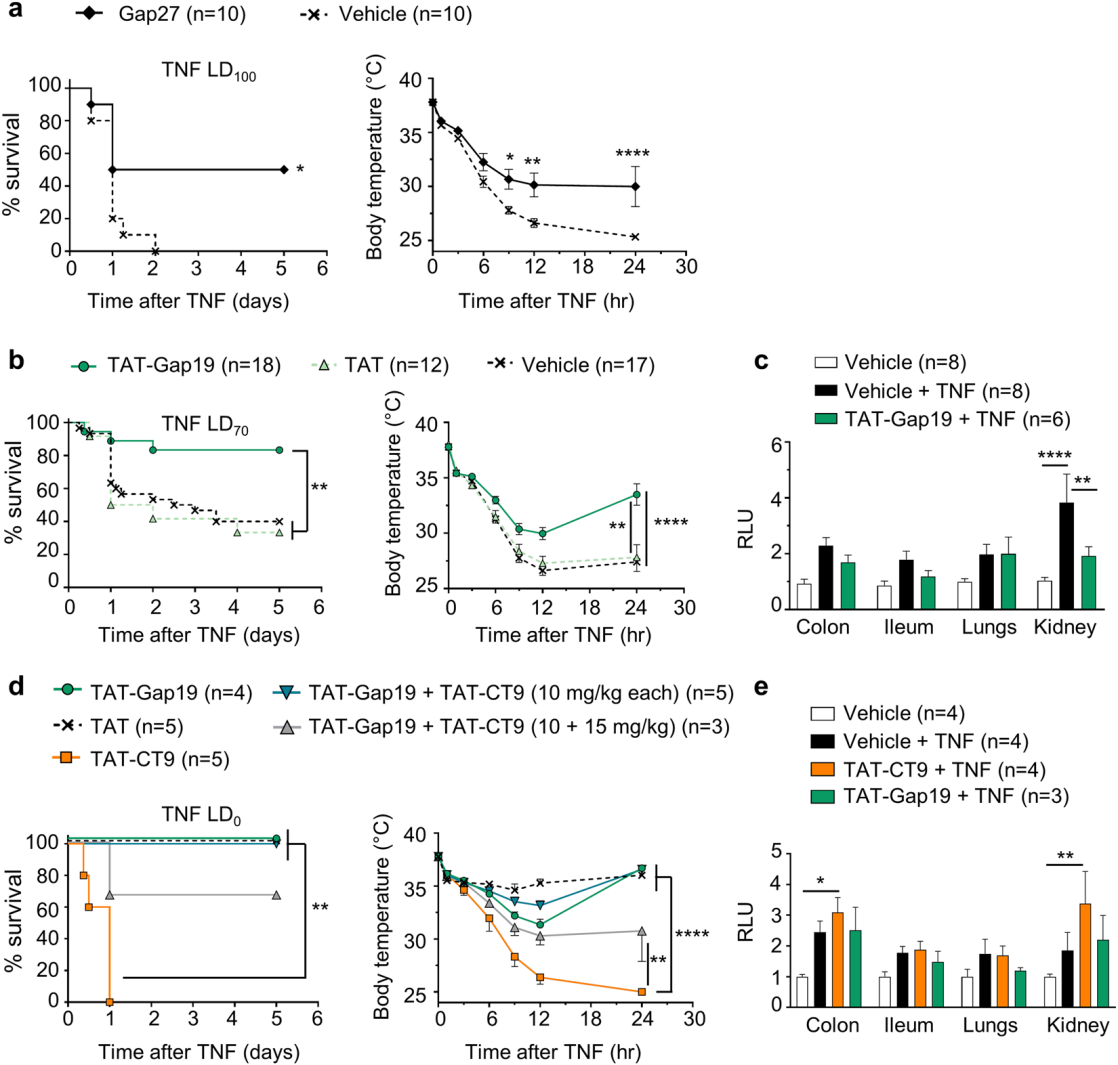
(**a**) Blocking Cx channels by Gap27 protects mice against TNF-induced mortality and hypothermia. Cumulative survival rates and body temperatures as a function of time. Male C57BL/6J mice were injected i.v. with Gap27 (25 mg/kg) or vehicle (DPBS) 15 min prior to i.v. injection of TNF (LD_100_). Pool of 2 independent experiments. The one-star differences in the left and right panel have p-values of 0.0212 and 0.0338, respectively; ^**^p = 0.0043, ^****^p < 0.0001. (**b,c**) Blocking Cx43 hemichannels protects mice against TNF-induced mortality, hypothermia and renal vascular permeability alterations. Male C57BL/6J mice were pre-injected i.v. with the indicated peptides 15 min prior to i.v. injection of TNF (LD_70_); n = total number of mice per group. **(b)** Cumulative survival rates and body temperatures presented as a function of time. Pool of 4 independent experiments. A significant protection is observed upon selective Cx43 hemichannel blockade by TAT-Gap19, when compared to pre-treatment with vehicle (^**^p = 0.0022^)^ or TAT (^**^p = 0.0067). The two-star difference (right panel) equals a p-value of 0.0019; ^****^p < 0.0001. LD_70_ = lethal dose for 70% of mice in control group (Vehicle + TNF). **(c)** Vascular permeability shown as relative light units (RLU) of FITC-dextran in colon, ileum, lungs and kidneys 6 hr after injection of vehicle or TNF. Pool of 2 independent experiments. The two-star difference corresponds to a p-value of 0.0073; ^****^p < 0.0001. **(d,e**) Stimulating Cx43 hemichannel opening accelerates TNF-induced mortality, hypothermia and vascular permeability increase. Male C57BL/6J mice were pre-injected i.v. with the indicated peptides 15 min prior to i.v. injection of a sub-lethal dose of TNF (LD_0_); n = total number of mice per group. **(d)** Survival and body temperature presented as a function of time. Mice receiving TAT-CT9 are sensitized towards TNF-induced lethality, when compared to mice pre-treated with TAT-Gap19 (^**^p = 0.0074), TAT (^**^p = 0.0034) and 10 mg/kg TAT-Gap19 + TAT-CT9 (^**^p = 0.0034) (d; left panel). The two-star difference in (d; right panel) corresponds to a p-value of 0.0078; ^****^p < 0.0001. LD_0_ = non-lethal dose for mice in control group (TAT + TNF). **(e)** Vascular permeability shown as RLU of FITC-dextran in colon, ileum, lungs and kidneys 6 hr after injection of vehicle or TNF. The one-star difference corresponds to a p-value of 0.0256, the two-star difference has a p-value of 0.0077. Data are presented as mean ± SEM. Statistical tests used: (**a** (left), **b** (left), **d** (left)): Mantel-Cox test; **a** (right): 2-Way ANOVA with post-hoc Sidak's test; (**b** (right), **c,d** (right), **e**): 2-Way ANOVA with post-hoc Tukey’s test.

The TNF-induced SIRS model was used to induce acute systemic inflammation, hypothermia and eventually death^1,4,24,25^. To address the role of Cx43 hemichannel opening, we injected TNF intravenously (i.v.) in C57BL/6J mice in the absence or presence of peptides modulating Cx43 hemichannel function. Prophylactic administration of Gap27 (25 mg/kg i.v.), a peptide mimicking a conserved domain on the second extracellular loop of various Cxs and known to first block hemichannels followed by delayed GJC block^26^, significantly protected 50% of the mice against TNF-induced hypothermia and mortality (Fig. 1a). To more specifically target Cx43 hemichannels, we prophylactically administered TAT-Gap19^19^ (5 mg/kg i.v.) and obtained significant protection against hypothermia and mortality (Fig. 1b). Injecting TAT-Gap19 30 min after TNF also significantly protected mice against hypothermia, and led to a smaller but significant reduction in mice mortality (17% vs 50% when administered prophylactically) (Supplemental Fig. 1a,b). Since vascular hyperpermeability is a major pathophysiological feature of SIRS^3,27^, we assessed the integrity of the endothelial barrier. For this, FITC-dextran was administered i.v. 5 hr post-TNF, and was removed from the blood vessels via transcardial perfusion, after which different organs were isolated to assess overall FITC-dextran leakage (Supplemental Fig. 2a). A significant increase in vascular permeability in colon, ileum, lungs and kidneys was detected 6 hr post-TNF injection (Supplemental Fig. 2b). Interestingly, blocking Cx43 hemichannels by TAT-Gap19 (5 mg/kg) impeded the TNF-induced increase in vascular permeability in the kidney (Fig. 1c), suggesting that Cx43 hemichannels open upon TNF injection, and thus contribute to vascular leakage during SIRS.

### Stimulating Cx43 hemichannel opening accelerates TNF-induced mortality, hypothermia and vascular permeability increase

To assess whether increased hemichannel opening would worsen lethality and vascular permeability, TAT-CT9 peptide that mimics part of the C-terminal tail of Cx43 and promotes Cx43 hemichannel opening, was used^20,22^. When mice were challenged with sub-lethal doses of TNF, TAT-CT9 (10 mg/kg i.v.) sensitized mice to TNF, leading to 100% mortality and stronger hypothermia (Fig. 1d) and furthermore significantly increased vascular permeability in the kidney, when compared to vehicle-treated mice (Fig. 1e). Interestingly, when both TAT-Gap19 and TAT-CT9 were combined at equimolar concentrations (10 mg/kg), TAT-Gap19 counteracted the sensitization effect of TAT-CT9, while increasing the TAT-CT9 concentration to 15 mg/kg, combined with 10 mg/kg TAT-Gap19, again increased hypothermia and mortality (Fig. 1d).

### TAT-Gap19 protection against renal vascular leakage is not accompanied by a reduction in cytokine and chemokine production, tissue damage, and release of cell death markers

**Figure 2:**
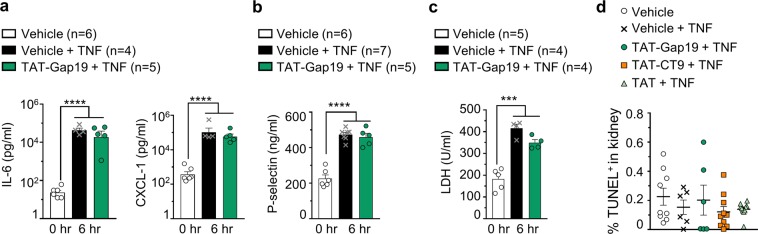
TAT-Gap19 protection against renal vascular leakage is not accompanied by a reduction in cytokine and chemokine production, tissue damage, and release of cell death markers. (**a–c)** Male C57BL/6J mice were pre-treated i.v. with vehicle (DPBS) or TAT-Gap19 (5 mg/kg) 15 minutes prior to TNF injection (10 µg, i.v.). n = total number of mice per group. **(a)** Levels of IL-6 and CXCL-1 in plasma of mice, 6 hr after TNF. **(b)** EC activation, displayed as plasma P-selectin levels 6 hr after TNF. **(c)** Tissue damage presented as levels of LDH in serum of mice, 6 hr after TNF. **(d)** Vehicle, TAT-Gap19 (10 mg/kg), TAT-CT9 (10 mg/kg) or TAT (10 mg/kg) were injected 15 min prior to TNF injection (10 µg, i.v.). Kidneys were isolated 6 hr after TNF. The data are represented as the ratio of TUNEL positive cells on all cells (Hoechst^+^) (in %). n = 5 mice per group; 1–3 sections per mouse; ^***^p < 0.0003, ^****^p < 0.0001. All data are presented as mean ± SEM. Statistical tests used: (a–d) 1-Way ANOVA with post-hoc Tukey’s test.

Since excessive Cx43 hemichannel opening may disturb the transmembrane balance of various ions and molecules, we investigated whether open hemichannels may lead to cell death^28^. To examine this end-point and the overall inflammatory state, the circulating levels of lactate dehydrogenase (LDH), reporting cell lysis, and pro-inflammatory cytokines and chemokines such as IL-6 and CXCL-1 were analysed 6 hr post-TNF. Increased levels of LDH, IL-6 and CXCL-1, as well as of P-selectin, marking EC activation, were observed (Fig. 2a–c). However, TAT-Gap19 (5 mg/kg) had no significant effect on these parameters (Fig. 2a–c). We further examined renal cell death by TUNEL assays, and surprisingly found no significant increase in TUNEL positivity following TNF compared to vehicle, suggesting TNF does not induce cell death in the kidneys 6 hr after TNF (Fig. 2d). Moreover, interfering with hemichannels by TAT-Gap19 or TAT-CT9 did not influence renal cell death levels after TNF (Fig. 2d). These data show that cell death is not the major cause for the increased renal vascular permeability, and that Cx43 hemichannels are not directly acting on the inflammatory and/or cell death cascades.

### TNF activates and promotes Cx43 hemichannel opening in a Ca^2+^-dependent manner

**Figure 3:**
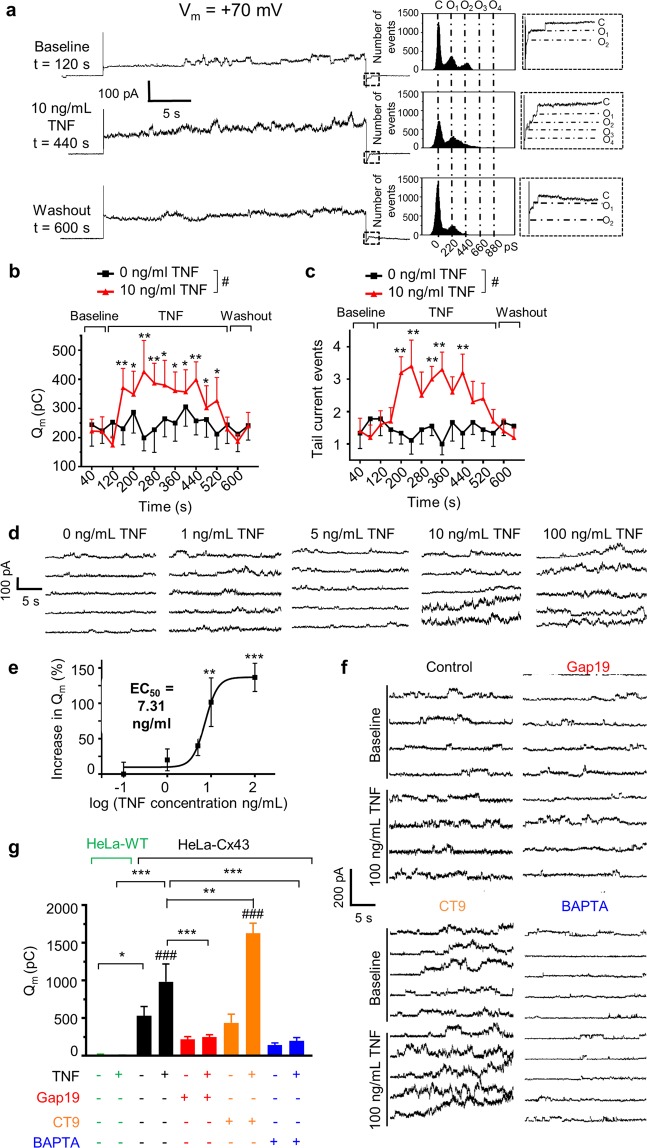
TNF promotes opening of Cx43 hemichannels in a Ca^2+^-dependent manner. (**a)** Example traces and matching all-point histograms depicting typical V_m_-induced (+70 mV, 30 s) Cx43 hemichannel unitary current activity recorded in solitary HeLa-Cx43 cells before, during and after application of human TNF (10 ng/ml) via a fast local perfusion system. Histograms illustrate conductance distributions centred around ~220 pS and multiples thereof. Dashed boxes right of the histograms correspond to boxed areas shown on the traces, demonstrating unitary channel closing events (conductance jumps of ~220 pS) in the tail currents following repolarization. **(b)** Q_m_ summary data at baseline, during 0 ng/ml (black trace) or 10 ng/ml acute TNF (red trace) exposure and following washout (n_cells_ = 10; 6 independent experiments). ‘#’ indicates significant difference in Q_m_ during 0 and 10 ng/ml TNF. **(c)** Graph as shown in (**b)** but based on tail current unitary event counting as a measure channel activity (n and # as in b). **(d)** Representative current traces for different TNF concentrations. **(e)** Dose-response relationship for TNF stimulation of unitary current activity (n_cells_ = 10 per concentration; 6 independent experiments). Different concentrations were compared to 0 ng/ml and fitted to a sigmoidal function. **(f)** Representative current traces illustrating the effect of Gap19, CT9 or BAPTA in baseline and in the presence of 100 ng/ml TNF (100 µM Gap19, 100 µM CT9 and 10 mM BAPTA intracellularly applied through the patch pipette). **(g)** Q_m_ summary data of (**f**) (n_cells_ = 15 per condition; 5 independent experiments). ‘#’ indicates significance as compared to baseline condition. ‘*’ indicates significance between conditions indicated by brackets. In (**b,c,e,g**), data are presented as mean ± SEM. Statistical tests used: (**b,c**) Repeated measures ANOVA with Dunnett’s post-hoc test; (**e**) 1-Way ANOVA and Bonferroni post-hoc test; (**g**) 2-tailed paired t-test or 1-Way ANOVA with Bonferroni post-hoc test, where applicable.

As TAT-Gap19 and TAT-CT9 respectively led to protection and sensitisation against hypothermia, mortality and permeability *in vivo*, we further analysed the effect of TNF on Cx43 hemichannel function using whole-cell voltage clamp on HeLa cells stably overexpressing Cx43^14,19,29^ and EA.hy926 human ECs. In HeLa-Cx43 cells, voltage steps to +70 mV provoked unitary currents with a unitary conductance (γ_0_) of ~220 pS, consistent with the opening of Cx43 hemichannels (Fig. 3a).

Within 40 s after application of 10 ng/ml TNF, the unitary currents doubled in associated membrane charge transfer, Q_m_, and the number of hemichannel closing events in the tail currents (Fig. 3a–c). Upon TNF washout, unitary currents reverted to baseline within 40 s; control experiments without TNF or with TNF on HeLa-wildtype (WT) cells had no effect (Supplemental Fig. 3a,b). TNF dose-dependently increased unitary currents with a maximum (~4-fold increase) at 100 ng/ml TNF, and a half-maximal effect (EC_50_) at ~7.31 ng/ml TNF (Hill coefficient = 3) (Fig. 3d,e). Moreover, Gap19 and CT9 (100 µM) respectively blocked or enhanced TNF-potentiated Cx43 hemichannel activity (Fig. 3f,g). TNF is known to provoke changes of intracellular Ca^2+^ concentration ([Ca^2+^]_i_)^30,31^, that may modulate Cx43 hemichannel opening^32^. Accordingly, Ca^2+^-imaging experiments on HeLa-Cx43 cells demonstrated Ca^2+^-oscillations in response to 100 ng/ml TNF (Supplemental Fig. 3c,d), and the TNF-induced increase in Q_m_ was prevented by the intracellular application of the Ca^2+^-chelator BAPTA (10 mM) (p < 0.001) (Fig. 3f,g).

**Figure 4:**
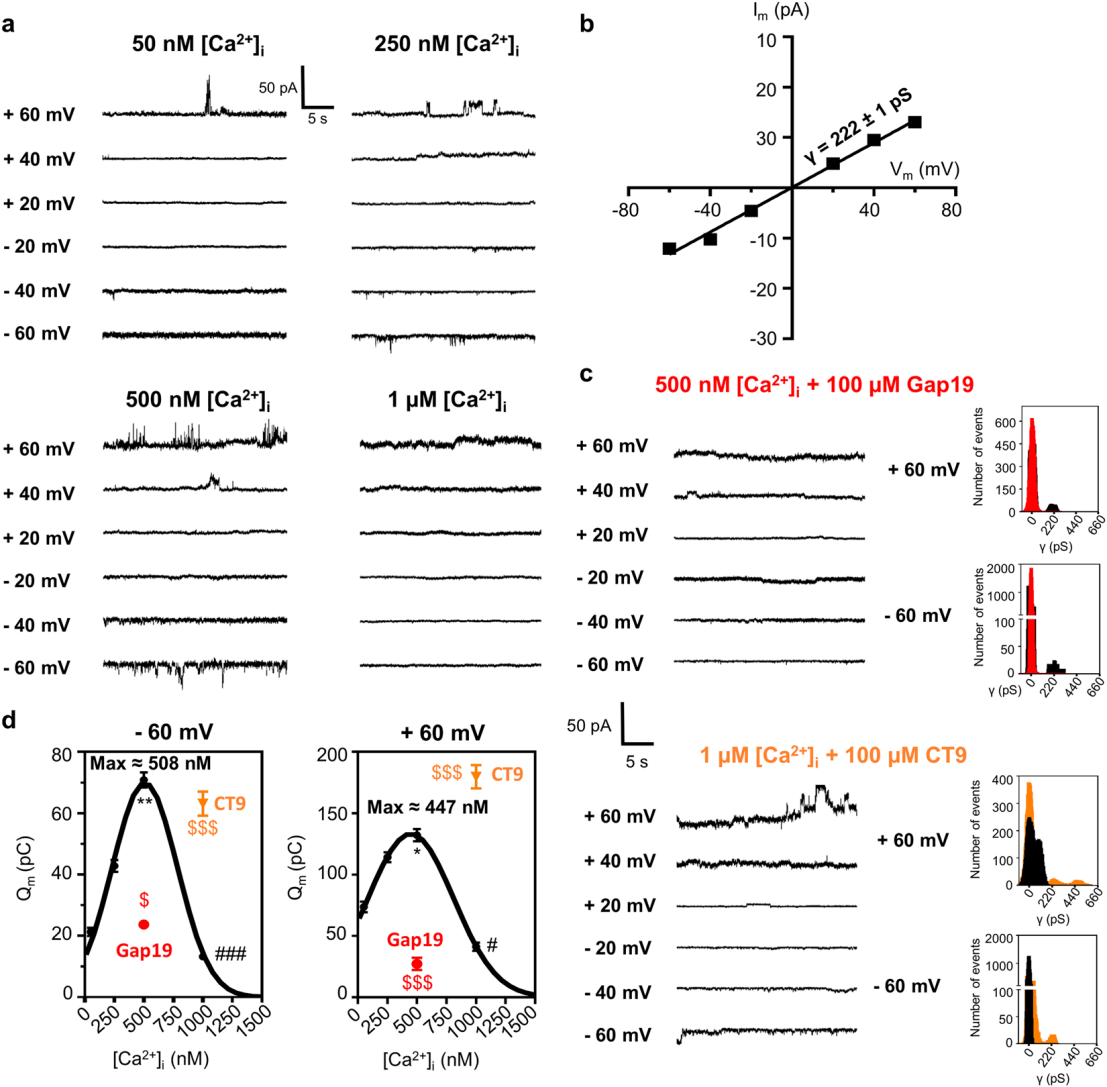
V_m_- and [Ca^2+^]_i_-dependent activation of Cx43 hemichannels in EA.hy926 cells. (**a)** Example traces depicting unitary current activities during 30 s incremental V_m_ steps (−60 to +60 mv in 20 mV increments) for different (50, 250, 500 and 1000 nM) [Ca^2+^]_i_ in solitary EA.hy926 cells. Increasing [Ca^2+^]_i_ to 250 or 500 nM clearly activated unitary current activities at negative V_m_ and promoted unitary current activities at positive V_m_; unitary currents disappear at 1 µM [Ca^2+^]_i_. **(b)** I-V plot of unitary current activities from experiments shown in **a**, demonstrating a slope conductance of 222 ± 1 pS (N_independent experiments_ = 6; n_cells_ = 21) characteristic for Cx43 hemichannels. **(c)** Respresentative current traces and corresponding all-point histograms at 500 or 1000 nM [Ca^2+^]_i_ with, respectively, 100 µM Gap19 or CT9 (control traces see 500 nM [Ca^2+^]_i_ panel a). **(d)** Q_m_ summary data for incremental V_m_ step protocols as illustrated in **a** and **c** (N_independent experiments_ = 6; n_cells_ = 11–24 per condition), showing increased hemichannel activity with 250 and 500 nM [Ca^2+^]_i_ and decreased activity at 1 µM. Gap19 (100 µM) blocked hemichannel activity at 500 nM [Ca^2+^]_i_ and CT9 enhanced hemichannel activity at 1 µM [Ca^2+^]_i_. Data are expressed as mean ± SEM. Comparisons were performed using a one-way ANOVA with bonferroni post-hoc test (for [Ca^2+^]_i_ dependency) or two-tailed unpaired t-test for Gap19 or CT9 versus 500 nM or 1µM [Ca^2+^]_i_. ‘*’ indicates significance of 500 nM [Ca^2+^]_i_ vs 50 nM [Ca^2+^]_i_. ‘#” indicates significance of 1 µM [Ca^2+^]_i_ vs 500 nM [Ca^2+^]_i_. ‘$’ indicates significance for Gap19 or CT9 versus respective control condition.

**Figure 5:**
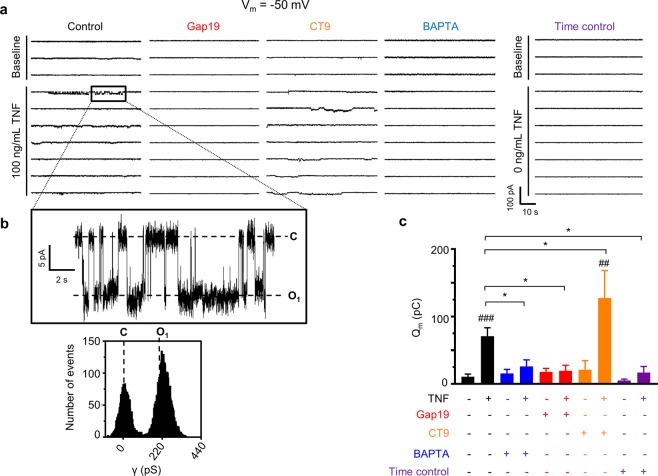
TNF rapidly activates Cx43 hemichannel opening in EA.hy926 cells via a Ca^2+^-dependent mechanism. (**a**) Consecutive current traces obtained at −50 mV (60 s) with and without 100 ng/mL TNF in solitary EA.hy926 cells. TNF induced periodic burst opening of ~220 pS unitary current activity, which was blocked by 100 µM Gap19 or 10 mM BAPTA, and enhanced by 100 µM CT9 (applied via the pipette solution). **(b)** Inset with corresponding all-point histogram demonstrating unitary activity characterized by a ~220 pS conductance. **(c)** Q_m_ summary data of **a** (n_cells_ = 8–13 per condition; 6 independent experiments). Data are expressed as mean ± SEM. ‘#’ indicates significance as compared to baseline condition, two-tailed paired student t test was used. ‘*’ indicates significance versus control TNF recording (2^nd^ bar), one-way ANOVA with Dunnett’s multiple comparison test was used.

We next tested in EA.hy926 ECs whether TNF could induce hemichannel opening by itself without electrical stimulation and whether [Ca^2+^]_i_ played a role in activation. At normal resting 50 nM [Ca^2+^]_i_, unitary currents only appeared at +60 mV but at higher [Ca^2+^]_i_ (250 or 500 nM), unitary currents additionally appeared at more physiological negative membrane potential (Fig. 4a). Upon increasing [Ca^2+^]_i_ further to 1 µM, hemichannel activities again decreased to the level observed at 50 nM (Fig. 4a). A plot of unitary current amplitudes at 500 nM [Ca^2+^]_i_, as a function of voltage was characterized by a slope conductance of 222 ± 1 pS, supporting Cx43 hemichannel activity (Fig. 4b). Plotting Q_m_ as a function of [Ca^2+^]_i_ gave a bell-shaped relation with high hemichannel activity at 500 nM [Ca^2+^]_i_ and low activity at 50 nM and 1 µM for both negative and positive voltages (Fig. 4d). Gap19 abolished hemichannel activity at 500 nM [Ca^2+^]_i_ and CT9 enhanced low hemichannel activity at 1 µM [Ca^2+^]_i_ (Fig. 4c,d). Histogram analysis of unitary currents demonstrated a single-channel conductance of ~220 pS for both control and CT9 (Fig. 4c). Collectively, these observations demonstrate [Ca^2+^]_i_-controlled Cx43 hemichannel opening^20,33^. Finally and most importantly, challenging EA.hy926 cells held at −50 mV resting potential with 100 ng/mL TNF triggered unitary currents with a ~220 pS unitary conductance, which were enhanced by CT9, and abolished by Gap19 and BAPTA [Ca^2+^]_i_-buffering, demonstrating Ca^2+^-dependent activation (Fig. 5a–c).

### Ca^2+^ and CaMKII are implicated in TNF-induced renal vascular permeability

**Figure 6:**
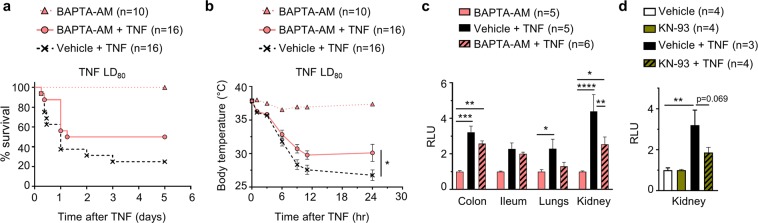
Ca^2+^ and CaMKII are implicated in TNF-induced renal vascular permeability. (**a–c)** Male C57BL/6J mice were pre-injected i.v. with vehicle (6% DMSO in DPBS) or BAPTA-AM (2 mM in DPBS) 30 min prior to i.v. injection of TNF (LD_80_); control condition is BAPTA-AM injected (i.v., 2 mM) without TNF; n = total number of mice per group. **(a,b)** Cumulative survival rates and body temperatures presented as a function of time. Pool of 3 independent experiments; ^*^p = 0.0324. **(c)** Vascular permeability shown as RLU of FITC-dextran in colon, ileum, lungs and kidneys 6 hr after injection of control (BAPTA-AM) or TNF. Pool of 2 independent experiments. Colon: ^**^p = 0.0086, ^***^p = 0.0003; Lungs: ^*^p = 0.0469; Kidney: ^****^p < 0.0001, ^*^p = 0.0108; ^**^p = 0.0017. (**d)** Male C57BL/6J mice were injected i.v. with vehicle (6% DMSO in DPBS) or KN-93 (10 mg/kg) 60 min prior to i.v. injection of TNF. Mice pre-treated with KN-93 are slightly (p = 0.069) protected against renal vascular leakage. The two-star difference equals a p-value of 0.0034. In (**b–d**), data are presented as mean ± SEM. The statistical tests used: (**a**) Mantel-Cox test; (**b,c**) 2-Way ANOVA with post-hoc Tukey’s test; (**d**) 1-Way ANOVA with post-hoc Tukey’s test.

Next, we investigated the role of intracellular Ca^2+^ in TNF-induced SIRS *in vivo* by [Ca^2+^]_i_ chelation with ester-loaded BAPTA-AM (2 mM i.v.)^34^, and found that it did not protect mice against mortality, but significantly ameliorated body temperatures (Fig. 6a,b), and protected against renal vascular leakage (Fig. 6c).

**Figure 7:**
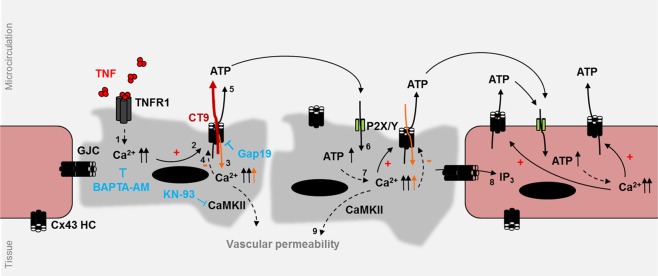
TNF-induced cytoplasmic changes in Ca^2+^ signaling with subsequent hemichannel opening contributes to vascular disruption. Schematic overview of TNF-induced Ca^2+^ changes^**1**^, leading to hemichannel opening in ECs**²** and other cells, further promoting Ca^2+^-entry^**3**^ (orange). High intracellular Ca^2+^ concentrations (>500 nM) negatively regulate hemichannel opening, providing a negative feedback^**4**^ (orange) (reviewed in^23^). Hemichannel opening is also associated with ATP-release^**5**^, which activates purinergic signaling through P2X-and P2Y-receptors^**6**^, followed by the spreading of cytoplasmic Ca^2+^ alterations to neighboring cells^**7**^. Intercellular movement of IP_3_ through GJCs^**8**^ may contribute to this spreading (reviewed in^38^). Ca^2+^ signaling is implicated in CaMKII-activation and auto-phosphorylation, and subsequent endothelial barrier integrity^**9**^. Both Ca^2+^-buffering by BAPTA-AM, CaMKII inhibition by KN-93 and blocking of Cx43 hemichannels by TAT-Gap19 (blue) protect cells against vascular leakage, while TAT-CT9 (dark red) promotes hemichannel opening and thus accelerates leakage.

Open Cx43 hemichannels form a release pathway for ATP^35^, which may subsequently activate autocrine or paracrine signaling via purinergic P2X- and P2Y-receptors^36^, thereby inducing Ca^2+^-increases in nearby cells that may provoke a propagation of hemichannel opening^37^. We thus tested whether P2X-/P2Y-receptor interference with PPADS, a broad-spectrum P2 receptor antagonist conferred protection against TNF-induced SIRS. PPADS (50 mg/kg i.p.) was found to significantly protect against hypothermia, visible after 6 hr and maintained until 24 hr, and against mortality (Supplemental Fig. 4). In a second instance, we assessed the role of CaMKII during TNF-induced SIRS as it is a known modulator of endothelial barrier integrity^38^. We found that the CaMKII inhibitor KN-93 (10 mg/kg i.v.) decreased TNF-induced renal vascular leakage, suggesting that CaMKII may be implicated in this process (Figs 6d and 7).

## Discussion

Our results indicate involvement of Cx43 hemichannels in TNF-induced SIRS pathology. First, TAT-Gap19 significantly protected mice against mortality, hypothermia and renal vascular permeability, while stimulating hemichannel opening with TAT-CT9 exhibited opposite effects and sensitized mice to TNF. Electrophysiological evidence demonstrated a fast stimulatory effect of TNF on Cx43 hemichannel opening, which was linked to [Ca^2+^]_i_-dynamics. *In vivo*, [Ca^2+^]_i_-buffering with BAPTA-AM also protected against renal vascular leakage and hypothermia, and we identified ATP and CaMKII as possible intermediate actors in triggering renal vascular leakage. Collectively, these data assign a new role to Cx43 hemichannels in accelerating renal vascular permeability and mortality during TNF-induced SIRS.

Cx43 channels have previously been implicated in vascular permeability alterations based on *in vivo* observations under inflammatory or ischemic conditions^39–41^ based on work with extracellular loop mimetic peptides, such as Peptide-5, Gap26 or Gap27^11^. However, these peptides may  affect various Cx isotypes distinct from Cx43 which is a prototypic Cx involved in inflammation. Similarly, Zhang *et al*. reported a relationship between Cx43 and renal permeability in response to cecal-ligation and puncture (CLP), making use of carbenoxolone^39^, which inhibits both GJCs and hemichannels, but also affects many other targets^42,43^. Here, we demonstrated a significant impact of selective Cx43 hemichannel blockade via TAT-Gap19 on renal vascular permeability. Other studies linked an increased *ex vivo* Cx43 expression in rat kidneys upon lipopolysaccharide treatment to a pro-inflammatory effect^44^, and showed an upregulation of Cx43 in chronic kidney disease, which was associated with increased pro-inflammatory signalling^45^. However, in these studies, Cx43 hemichannel functionality was not addressed.

Circulating peptides are rapidly cleared from the blood suggesting that the protective effect of TAT-Gap19 on mortality occurring over a time span of several days involves an early and crucial event. Of interest, in a model of mild CLP in Balb/c mice, Gap26 and TAT-Gap19 exhibited opposite effects, with Gap26 being protective and TAT-Gap19 worsening mortality^46^. However, the TAT-Gap19 doses used were very high (up to 120 mg/kg compared to 5 mg/kg in this study).

TAT-CT9, which enhances hemichannel opening^20,21^, was used to contrast the TAT-Gap19 effects, and was shown to sensitize the TNF-induced phenotype *in vivo*. To understand the opposite effects of TAT-Gap19 and TAT-CT9, we investigated the modulating effect of TNF on hemichannel activity through electrophysiological recording and found that TNF rapidly and dose-dependently stimulated Cx43 hemichannel opening, via [Ca^2+^]_i_-elevation. These findings demonstrate that Cx43 hemichannel opening is an early event that may crucially affect the outcome, as exemplified by the strong protective effect of a single injection of TAT-Gap19 on long lasting outcome measurements such as mortality.

It is well known that Cx hemichannels are also involved as a pathway for ATP-release^35,47^, which subsequently induces [Ca^2+^]_i_ -elevation in neighbouring cells via paracrine purinergic signalling^48,49^. In fact, hemichannel-linked ATP-release plays a major role in cell-to-cell propagation of Ca^2+^-waves^37^, which have been demonstrated to facilitate spread of inflammation along the vascular endothelium. Interestingly, PPADS protected mice against TNF-induced mortality and hypothermia, suggesting that purinergic receptor activation is contributing to TNF-induced pathology. BAPTA-AM on the other hand only protected mice against TNF-induced hypothermia and renal vascular permeability, but could not rescue against mortality, presumably because BAPTA-AM does not only inhibit pathological Ca^2+^-signalling, but also interferes with normal cell function. As a result, organ function may be disturbed by BAPTA-AM thereby compromising the function of heart, liver, kidney, brain and others, which may tip the mortality balance and obscure the protective potential.

The causal relation between vascular permeability and mortality is still a matter of debate^8^. However, different strategies for ameliorating microvascular leakage in several mouse models of sepsis were associated with better survival outcome^8^. Interestingly, these protective effects were not associated with lower inflammatory cytokine levels, as found here and in another study, investigating the role of receptor interacting protein kinase (RIPK) 1-dependent necroptosis in TNF-induced SIRS^3^. Although the mechanisms by which vascular permeability is induced are still not fully understood, it is conceivable that excessive opening of hemichannels upon TNF-induced changes in [Ca^2+^]_i_ could lead to detrimental outcomes in cells, via a cellular imbalance of ionic/molecular gradients^17,18^ and a subsequent osmotic water flow^50^. Indeed, the contribution of open hemichannels to cell death induction has been debated in various studies^28,41,46^ and has been reported in retinal ECs under ischemic conditions^41^. However, in our study, the TNF challenge did not promote renal cell death, as quantified by TUNEL staining, suggesting it was not causative for the observed TNF-induced renal permeability. In contrast, in the liver, TNF-induced endothelial permeability was shown to be induced by RIPK1-dependent necroptosis^3^. Another pathway underlying permeability can be attributed to Ca^2+^ and CaMKII function^38^. CaMKII has been linked to myosine light chain (MLC)- and MLC kinase-phosphorylation^51^, which are importantly involved in vascular endothelial barrier dysfunction^38,39,52,53^. Our finding of decreased renal vascular leakage with KN-93 CaMKII inhibition points to involvement of a Ca^2+^/CaMKII-dependent cascade, which is a central hub in the signalling cascade leading to endothelial barrier leakage^38^.

These findings emphasize the specific contribution of Cx43 hemichannels in inducing vascular permeability and blocking these channels protects against TNF-induced vascular leakage, hypothermia and eventually mortality. Limitations of the present study are that the peptides used have a short life-time and low *in vivo* efficacy and that the TNF-induced SIRS mice model has limited relevance for the complex condition of human SIRS/sepsis. As a result, further studies are needed to unravel the potential role of Cx43 targeted therapies in the clinical setting of SIRS treatment.

## Materials and Methods

### Cells

HeLa cells, stably transfected with Cx43^19,29,54^, were cultured in Dulbecco’s modified eagle medium (Invitrogen, Ghent, Belgium), supplemented with 10% fetal bovine serum, 1% sodium pyruvate, 2 mM glutamine, 10 µg/ml streptomycin, 10 U/ml penicillin, 0.25 µg/ml fungizone (Invitrogen, Ghent, Belgium) and 1 µg/ml puromycin (Sigma-Aldrich, Bornem, Belgium). HeLa-WT cells were grown in the medium without puromycin. Mouse *GJA1* gene was cloned into the *Eco*RI/*Bam*HI restricted cloning site of the expression vector pMJgreen. Cytomegalovirus promoter was used. The vector also contains a puromycin N-acetyl-transferase (Pac) gene encoding region. EA.hy926 human ECs, a hybrid clone generated by fusing HUVECs with A549 cells, were cultured in DMEM, supplemented with 10% FBS, 1% L-glutamine, 0.4% sodium pyruvate and 2% HT-supplement (Invitrogen, Ghent, Belgium). All cells were cultured at 37 °C in a 10% CO_2_ atmosphere and were tested for mycoplasma on a regular basis.

### Reagents and antibodies

Recombinant mouse TNF and human TNF were produced in *Escherichia coli* and purified (>95%) in the VIB Protein Core (IRC, Ghent, Belgium). The biological activity of murine TNF (*in vivo*) and human TNF (*in vitro*) was 7.94 × 10^9^ IU/mg and 6.8 × 10^7^ IU/mg respectively, and was determined using an MTT [3-[4,5-dimethylthiazole-2-yl]-2,5-diphenyltetrazolium bromide] assay. Peptides (purity > 95%) were purchased from Pepnome (China) and Genosphere Biotechnologies (Paris, France). Endotoxin contamination was checked by a Limulus Amebocyte Lysate assay and was <1.0 EU/mg. Gap27 (SRPTEKTIFII), TAT-Gap19 (YGRKKRRQRRR-KQIEIKKFK), Gap19 (KQIEIKKFK), TAT-CT9 (YGRKKRRQRRR-RPRPDDLEI), CT9 (RPRPDDLEI) and TAT (YGRKKRRQRRR) were diluted in endotoxin-free phosphate-buffered saline (PBS) or pipette solution for *in vivo* or *in vitro* use respectively. BAPTA was purchased from Invitrogen (B1212). BAPTA-AM (Invitrogen, B6769) was dissolved in dimethylsulfoxide (DMSO) (+0.01% pluronic® (Invitrogen)) and further diluted in endotoxin-free PBS for *in vivo* use. Pyridoxalphosphate-6-azophenyl-2′,4′-disulfonic acid (PPADS, Sigma-Aldrich), and KN-93 (Calbiochem) were dissolved in DMSO and further diluted in endotoxin-free PBS for *in vivo* use. Fluorescein isothiocyanate (FITC)-dextran (4kDa; Sigma-Aldrich) was used in vascular permeability studies.

### Electrophysiological recording

Subconfluent cultures of HeLa-Cx43 cells and EA.hy926 cells were seeded on 13-mm-diameter glass coverslips (Knittel Glaser, Novolab, Geraardsbergen, Belgium) and experiments were performed at subconfluency the next day. All recordings were performed in the presence of extracellular Ca^2+^ and Mg^2+^ and under conditions of K^+^-channel blockade with Cs^+^, Ba^2+^ and TEA^+^. Cells were bathed in a recording chamber filled with a modified Krebs-Ringer solution, consisting of (in mM): 150 NaCl, 6 CsCl, 2 MgCl_2_, 2 CaCl_2_, 5 glucose (10 for EA.hy926 cells), 5 HEPES, 1 BaCl_2_ and 2 Pyruvate, with pH adjusted to 7.4. The standard whole-cell recording pipette solution was composed of (in mM): 130 CsCl, 10 NaAsp, 0.26 CaCl_2_, 5 HEPES, 2 EGTA, 5 TEA-Cl and 1 MgCl_2_, with pH adjusted to 7.2. For experiments on EA.hy926 cells, 5 mM MgATP was additionally added to the pipette solution. A baseline free intracellular Ca^2+^ ([Ca^2+^]_i_, applied through the whole cell recording pipette) of 50 nM, as well as other concentrations (250, 500 and 1000 nM), were calculated with Webmax Standard software (http://www.stanford.edu/~cpatton/webmaxcS.htm). In experiments where cells were challenged with TNF, the pipette EGTA concentration was 2 mM for HeLa-Cx43 cells and 0.1 mM for EA.hy926 cells; for Ca^2+^ chelation experiments aimed at suppressing [Ca^2+^]_i_ changes, EGTA was changed to 10 mM BAPTA. Gap19 and CT9 peptides (100 µM) were added to the pipette solution. Cells were exposed to recombinant human TNF via a fast local perfusion system. An EPC 7 PLUS patch-clamp amplifier (HEKA Elektronik, Lambrecht, Pfalz, Germany) was used for single channel recordings. Data were acquired at 6 kHz using a NI USB-6221 data acquisition device from National Instruments (Austin, TX, USA) and WinWCP acquisition software (designed by Dr. J. Dempster; University of Strathclyde, UK). All currents in whole-cell configuration were filtered at 1 kHz (7-pole Besselfilter). For single channel analysis, holding currents were subtracted from the recorded current traces, giving traces that only contained unitary current events. Unitary conductances were calculated from the elementary current transitions Δ*i* as: *γ* = Δ*i*/*V*_m_. From these data, we constructed all-point conductance histograms that displayed one or more Gaussian distributions. These were fit by a probability density function assuming independent channel opening^19,29,55,56^. Channel activity was quantified from the charge transfer *Q*_m_ associated with unitary currents; this was done by integrating the unitary current traces over the duration of the voltage step as: *Q*_m_ = ∫*i*d*t*. Unitary events in the tail currents were counted manually.

### Ca^2+^ imaging

HeLa-Cx43 cells were seeded onto 18-mm-diameter glass coverslips (Knittel Glaser, Novolab, Geraardsbergen, Belgium), and experiments were performed at subconfluency the next day. The cells were loaded with 5 µM fluo3-AM in HBSS-HEPES (1 mM CaCl_2_, 0.81 mM MgSO_4_, 13 mM NaCl, 0.18 mM Na_2_HPO_4_, 5.36 mM KCl, 0.44 mM KH_2_PO_4_, 5.55 mM d-glucose, and 25 mM HEPES) for 45 min at 37 °C. After 45 min, coverslips were washed and then left for an additional 15 min at 37 °C in HBSS-HEPES to allow for de-esterification. Cells were thereafter transferred to an inverted epifluorescence microscope (Eclipse TE 300, Nikon Belux, Brussels, Belgium) equipped with a superfusion system allowing fast solution changes. Superfusion was switched off during the registration of oscillatory activity. Images were taken every second with a 40x oil-immersion objective and an electron-multiplying CCD camera (Quantem 512SC, Photometrics, Tucson, AZ). We used a Lambda DG-4 filterswitch (Sutter Instrument Company, Novato, CA) to deliver excitation at 482 nm and captured emitted light via a 505-nm long-pass dichroic mirror and a 535-nm bandpass filter (35-nm bandwidth). Recordings and analysis were done with custom-developed QuantEMframes and Fluoframes software written in Microsoft Visual C^++^ 6.0. Oscillatory activity was recorded in a 10 min observation window.

### Mice

All mice were bred in specific pathogen-free or in conventional animal facilities, and were males between 8–12 weeks during all the experiments. As connexin expression levels can be altered by the hormonal cycle, male mice were used to limit variability related to the hormonal status. Mice were housed in air-conditioned, temperature-controlled rooms with 14/10-hour light/dark cycles. Food and water were provided *ad libitum*. C57BL/6J WT mice were purchased from Janvier (Le Genest, France). All mice experiments were organized in accordance with institutional, national, and European animal regulations, and got approved by the animal ethics committee at Ghent University (ECD 2013–064).

### TNF induced Lethal Shock – Monitoring and Sampling

Peptides were diluted in endotoxin-free PBS (Sigma-Aldrich) and injected i.v. in a volume of 200 µl at 5 or 10 mg/kg for TAT-Gap19, 5 or 10 mg/kg for TAT, 10 or 15 mg/kg for TAT-CT9, and 25 mg/kg for Gap27, 15 min before i.v. injection of murine TNF (7.5–15 µg/20 g) in 200 µl endotoxin-free PBS (pH 6.8) (prophylactic administration). Therapeutic administration consisted of i.v. injection of 5 mg/kg of TAT-Gap19 30 min after TNF. BAPTA-AM (2 mM in DPBS (6% DMSO)) and KN-93 (10 mg/kg in DPBS (6% DMSO)) were injected i.v. respectively 30 min and 60 min before TNF injection. Control injections consisted of an equal amount (i.v.) of DPBS (6% DMSO). PPADS (50 mg/kg) was injected intraperitoneally (i.p.) 15 min before injection of TNF. Survival and body temperatures were monitored until 5 days post TNF-injection. Rectal body temperatures were recorded with an electric thermometer (Model 1; Comark Electronics, Norwich, UK), and dead mice (25 °C) are included in all body temperature graphs, as was previously implemented^1,57^. Blood and tissue samples were collected at different time points after injection as outlined in the results section. Blood was collected by cardiac or retro-orbital puncture. Vascular permeability was evaluated as previously described^58^.

### Immunostaining

Tissue was fixed with 4% paraformaldehyde, embedded in paraffin, sectioned at 5 µm and dewaxed before staining procedure was initiated. Cell death was identified by terminal deoxynucleotidyl-transferase dUTP nick-end labeling (TUNEL) staining (TMR *in situ* cell death detection kit; Roche) in accordance with the manufacturer’s instructions, 6 hr after TNF injection. Sections were counterstained with Hoechst (Sigma-Aldrich). Micrographs were acquired using a Zeiss Axioscan Z.1 slide scanner (Carl Zeiss, Jenna, Germany) at 20x magnification, with a Hamamatsu ORCA Flash4 camera (Hamamatsu Photonics), and illuminated with an HXP 120V light source. Analysis was performed using Volocity^Ⓡ^ (PerkinElmer, UK) and QuPath (GitHub) softwares.

### Determination of LDH, Cytokines and P-selectin

Levels of LDH were measured by an automated clinical analyzer in the University Hospital of Ghent. Cytokine levels of IL-6 and chemokine (C-X-C motif) ligand 1 (CXCL-1) were determined with Luminex magnetic bead-based assays (R&D Systems, Biotechne). Plasma P-selectin levels were measured through a Quantikine ELISA kit (R&D Systems, Biotechne).

### Statistics

Statistical analysis was performed in GraphPad Prism (v.7.0). All Kaplan-Meier survival curves were analysed by the log-rank Mantel-Cox test. Body temperatures and vascular permeability assays were analysed by a two-way ANOVA with Tukey’s multiple comparisons test to compare more than two groups, and by a two-way ANOVA with Sidak’s multiple comparisons test to compare two groups. A Shapiro-Wilk normality test indicated normally distributed data. All immunohistochemical and serum parameters were compared with a one-way ANOVA with a Bonferroni post-test to compare all pairs. In the electrophysiology recordings, comparisons between two groups were done with a two-tailed t-test and comparison of more than two groups was performed with a one-way ANOVA and Bonferroni post-hoc test. Significant results are indicated with ^*^for p < 0.05; ^**^for p < 0.01; ^***^for p < 0.001 or ^****^for p < 0.0001.

## Supplementary information

Supplementary Figures and Legends

## Data Availability

All data generated or analysed during this study are included in this published article (and its Supplementary Information Files).

## References

[1] Duprez L (2011). RIP kinase-dependent necrosis drives lethal systemic inflammatory response syndrome. Immunity.

[2] Tracey KJ (1986). Shock and tissue injury induced by recombinant human cachectin. Science.

[3] Zelic M (2018). RIP kinase 1-dependent endothelial necroptosis underlies systemic inflammatory response syndrome. J Clin Invest.

[4] Newton K (2016). RIPK3 deficiency or catalytically inactive RIPK1 provides greater benefit than MLKL deficiency in mouse models of inflammation and tissue injury. Cell Death Differ.

[5] Goddard LM, Iruela-Arispe ML (2013). Cellular and molecular regulation of vascular permeability. Thromb Haemost.

[6] Clark PR, Kim RK, Pober JS, Kluger MS (2015). Tumor necrosis factor disrupts claudin-5 endothelial tight junction barriers in two distinct NF-kappaB-dependent phases. PLoS One.

[7] Kandasamy K, Escue R, Manna J, Adebiyi A, Parthasarathi K (2015). Changes in endothelial connexin 43 expression inversely correlate with microvessel permeability and VE-cadherin expression in endotoxin-challenged lungs. Am J Physiol Lung Cell Mol Physiol.

[8] Goldenberg NM, Steinberg BE, Slutsky AS, Lee WL (2011). Broken barriers: a new take on sepsis pathogenesis. Sci Transl Med.

[9] Decrock E (2009). Connexin-related signaling in cell death: to live or let die?. Cell Death Differ.

[10] Wang N (2013). Paracrine signaling through plasma membrane hemichannels. Biochim Biophys Acta.

[11] Delvaeye T, Vandenabeele P, Bultynck G, Leybaert L, Krysko D (2018). Therapeutic targeting of connexin channels: new views and challenges. Trends Mol Med.

[12] Laird DW (2006). Life cycle of connexins in health and disease. Biochem J.

[13] Trexler EB, Bennet MVL, Bargiello TA, Verselis VK (1996). Voltage gating and permeation in a gap junction hemichannel. Proc Natl Acad Sci USA.

[14] Contreras JE, Saez JC, Bukauskas FF, Bennett MV (2003). Gating and regulation of connexin 43 (Cx43) hemichannels. Proc Natl Acad Sci USA.

[15] Retamal MA (2007). Cx43 hemichannels and gap junction channels in astrocytes are regulated oppositely by proinflammatory cytokines released from activated microglia. J Neurosci.

[16] De Vuyst E (2007). Connexin hemichannels and gap junction channels are differentially influenced by Lipopolysaccharide and Basic Fibroblast Growth Factor. Mol Biol Cell.

[17] Saez JC (2010). Cell membrane permeabilization via connexin hemichannels in living and dying cells. Exp Cell Res.

[18] Retamal MA (2015). Diseases associated with leaky hemichannels. Front Cell Neurosci.

[19] Wang N (2013). Selective inhibition of Cx43 hemichannels by Gap19 and its impact on ischemia/reperfusion injury. Basic Res Cardiol.

[20] Bol M (2017). At the cross-point of connexins, calcium, and ATP: blocking hemichannels inhibits vasoconstriction of rat small mesenteric arteries. Cardiovasc Res.

[21] Retamal MA (2014). Opening of pannexin- and connexin-based channels increases the excitability of nodose ganglion sensory neurons. Front Cell Neurosci.

[22] Ponsaerts R (2010). Intramolecular loop/tail interactions are essential for connexin 43-hemichannel activity. FASEB J.

[23] Leybaert L (2017). Connexins in Cardiovascular and Neurovascular Health and Disease: Pharmacological Implications. Pharmacol Rev.

[24] Vanden Berghe T (2014). Simultaneous targeting of IL-1 and IL-18 is required for protection against inflammatory and septic shock. Am J Respir Crit Care Med.

[25] Delvaeye T (2018). Noninvasive whole-body imaging of phosphatidylethanolamine as a cell death marker using (99m)Tc-duramycin during TNF-induced SIRS. J Nucl Med.

[26] Wang N (2013). Connexin targeting peptides as inhibitors of voltage- and intracellular Ca2+-triggered Cx43 hemichannel opening. Neuropharmacology.

[27] Vandendriessche B (2014). MAPK-activated protein kinase 2-deficiency causes hyperacute tumor necrosis factor-induced inflammatory shock. BMC Physiol.

[28] Decrock E (2009). Connexin 43 hemichannels contribute to the propagation of apoptotic cell death in a rat C6 glioma cell model. Cell Death Differ.

[29] Wang N (2012). Connexin mimetic peptides inhibit Cx43 hemichannel opening triggered by voltage and intracellular Ca^2+^ elevation. Basic Res Cardiol.

[30] Duncan DJ, Yang Z, Hopkins PM, Steele DS, Harrison SM (2010). TNF-alpha and IL-1beta increase Ca2+ leak from the sarcoplasmic reticulum and susceptibility to arrhythmia in rat ventricular myocytes. Cell Calcium.

[31] De A, Krueger JM, Simasko SM (2003). Tumor necrosis factor α increases cytosolic calcium responses to AMPA and KCl in primary cultures of rat hippocampal neurons. Brain Res.

[32] De Vuyst E (2009). Ca(2+) regulation of connexin 43 hemichannels in C6 glioma and glial cells. Cell Calcium.

[33] De Bock M (2012). Connexin 43 hemichannels contribute to cytoplasmic Ca2+ oscillations by providing a bimodal Ca^2+^-dependent Ca^2+^ entry pathway. J Biol Chem.

[34] Tsien RY (1981). A non-disruptive technique for loading calcium biffers and indicators into cells. Nature.

[35] Kang J (2008). Connexin 43 hemichannels are permeable to ATP. J Neurosci.

[36] Albert JL (1997). Regulation of brain capillary endothelial cells by P2Y receptors coupled to Ca^2+^, phospholipase C and mitogen-activated protein kinase. Br J Pharmacol.

[37] Leybaert L, Sanderson MJ (2012). Intercellular Ca(2+) waves: mechanisms and function. Physiol Rev.

[38] Borbiev T, Verin AD, Shi S, Liu F, Garcia JGN (2001). Regulation of endothelial cell barrier function by calcium/calmodulin-dependent protein kinase II. Am J Physiol Lung Cell Mol Physiol.

[39] Zhang J (2015). Role of connexin 43 in vascular hyperpermeability and relationship to Rock1-MLC20 pathway in septic rats. Am J Physiol Lung Cell Mol Physiol.

[40] Parthasarathi K (2012). Endothelial connexin43 mediates acid-induced increases in pulmonary microvascular permeability. Am J Physiol Lung Cell Mol Physiol.

[41] Danesh-Meyer HV (2012). Connexin43 mimetic peptide reduces vascular leak and retinal ganglion cell death following retinal ischaemia. Brain.

[42] Vessey JP (2004). Carbenoxolone inhibition of voltage-gated Ca channels and synaptic transmission in the retina. J Neurophysiol.

[43] Lohman AW (2015). Pannexin 1 channels regulate leukocyte emigration through the venous endothelium during acute inflammation. Nat Commun.

[44] Fernandez-Cobo M, Gingalewski C, De Maio A (1998). Expression of the connexin 43 gene is increased in the kidneys and the lungs of rats injected with bacterial lipopolysaccharide. Shock.

[45] Toubas J (2011). Alteration of connexin expression is an early signal for chronic kidney disease. Am J Physiol Renal Physiol.

[46] Li W (2018). Connexin 43 Hemichannel as a Novel Mediator of Sterile and Infectious Inflammatory Diseases. Sci Rep.

[47] Lohman AW, Billaud M, Isakson BE (2012). Mechanisms of ATP release and signalling in the blood vessel wall. Cardiovasc Res.

[48] Stout CE, Costantin JL, Naus CC, Charles AC (2002). Intercellular calcium signaling in astrocytes via ATP release through connexin hemichannels. J Biol Chem.

[49] Pubill D (2001). ATP induces intracellular calcium increases and actin cytoskeleton disaggregation via P2x receptors. Cell Calcium.

[50] Rodriguez-Sinovas A (2007). The modulatory effects of connexin 43 on cell death/survival beyond cell coupling. Prog Biophys Mol Biol.

[51] Garcia JGN, Davis HW, Patterson CE (1995). Regulation of endothelial cell gap formation and barrier dysfunction: role of myosin light chain phosphorylation. J Cell Physiol.

[52] McKenzie JA, Ridley AJ (2007). Roles of Rho/ROCK and MLCK in TNF-alpha-induced changes in endothelial morphology and permeability. J Cell Physiol.

[53] O’Donnell JJ, Birukova AA, Beyer EC, Birukov KG (2014). Gap junction protein connexin43 exacerbates lung vascular permeability. PLoS One.

[54] Elfgang C (1995). Specific permeability and selective formation of gap junction channels in connexin-transfected HeLa cells. J Cell Biol.

[55] Ramanan SV, Brink PR (1993). Multichannel recordings from membranes which contain gap junctions. Biophys J.

[56] Wang H (2001). Intercellular communication in cultured human vascular smooth muscle cells. Am J Physiol Cell Physiol.

[57] Cauwels A (2009). Nitrite protects against morbidity and mortality associated with TNF- or LPS-induced shock in a soluble guanylate cyclase-dependent manner. J Exp Med.

[58] Vandenbroucke RE (2012). Matrix metalloprotease 8-dependent extracellular matrix cleavage at the blood-CSF barrier contributes to lethality during systemic inflammatory diseases. J Neurosci.

