# Supplementary material for: Blocking connexin43 hemichannels protects mice against tumour necrosis factor-induced inflammatory shock
Source: Sci Rep. 2019 Nov 12;9:16623. doi: 10.1038/s41598-019-52900-4 (PMC6851386; doi:10.1038/s41598-019-52900-4)
Supplement: Supplementary file 1 — Supplementary Figures and Legends [file 41598_2019_52900_MOESM1_ESM.pdf]

Title: Blocking connexin43 hemichannels protects mice against tumour necrosis factor-induced inflammatory shock

Tinneke Delvaeye[1,2,3], Maarten A.J. De Smet[3], Stijn Verwaerde[3], Elke Decrock[3], Aleksandra Czekaj[1,2], Roosmarijn E. Vandenbroucke[1,2], Kelly Lemeire[1,2], Amanda Gonçalves[1,2,4], Wim Declercq[1,2], Peter Vandenabeele[1,2,5,#,*], Dmitri V. Krysko[6,#,*], Luc Leybaert[3,#,*]

Supplementary Figures and Legends

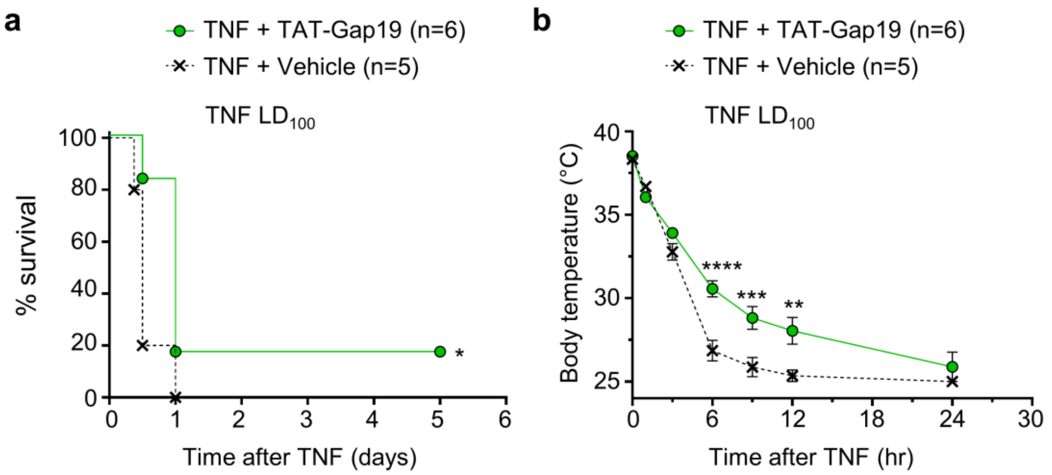

Supplemental Fig. 1: (a-b) TAT-Gap19 still protects mice against mortality when administered 30 minutes after TNF. Survival rate (a) and body temperature (b) as a function of time. Male C57BL/6J mice were injected i.v. with TAT-Gap19 (5 mg/kg) or vehicle (DPBS) 30 min after i.v. injection of TNF (LD_{100}). The one-star difference in the left panel corresponds to a p-value of 0.0348; $^{****}p<0.0001$, $^{***}p=0.001$, $^{**}p=0.003$. LD_{100} = lethal dose for 100 % of mice in control group (Vehicle + TNF). n = total number of mice per group. In (b), data are presented as mean ± SEM. Statistical tests used: (a) Mantel-Cox test; (b) 2-Way ANOVA with post-hoc Sidak's test.

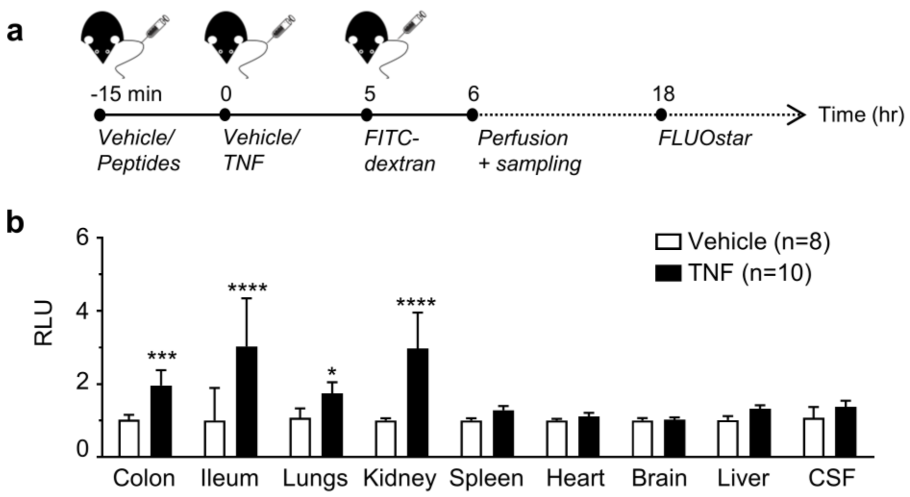

Supplemental Fig. 2: TNF-induced SIRS causes severe vascular leakage. (a) The vascular permeability assay used *in vivo* in Figures 1c, 1e, 6c and 6d. **(b)** Vascular permeability shown as relative light units (RLU) of FITC-dextran in different organs 6 hr after injection of vehicle (DPBS) or TNF (15 µg/20 g, i.v.). Two independent experiments; *p=0.0308; ***p=0.0002; ****p<0.0001. CSF = cerebrospinal fluid. n = total number of mice per group. In (b), data are presented as mean ± 95% CI. Statistical test used: (b) 2-Way ANOVA with post-hoc Tukey's test. *("Rat" icon by N.K. Narasimhan and "Syringe" icon by Ricardo Moreira from www.thenounproject.com)*

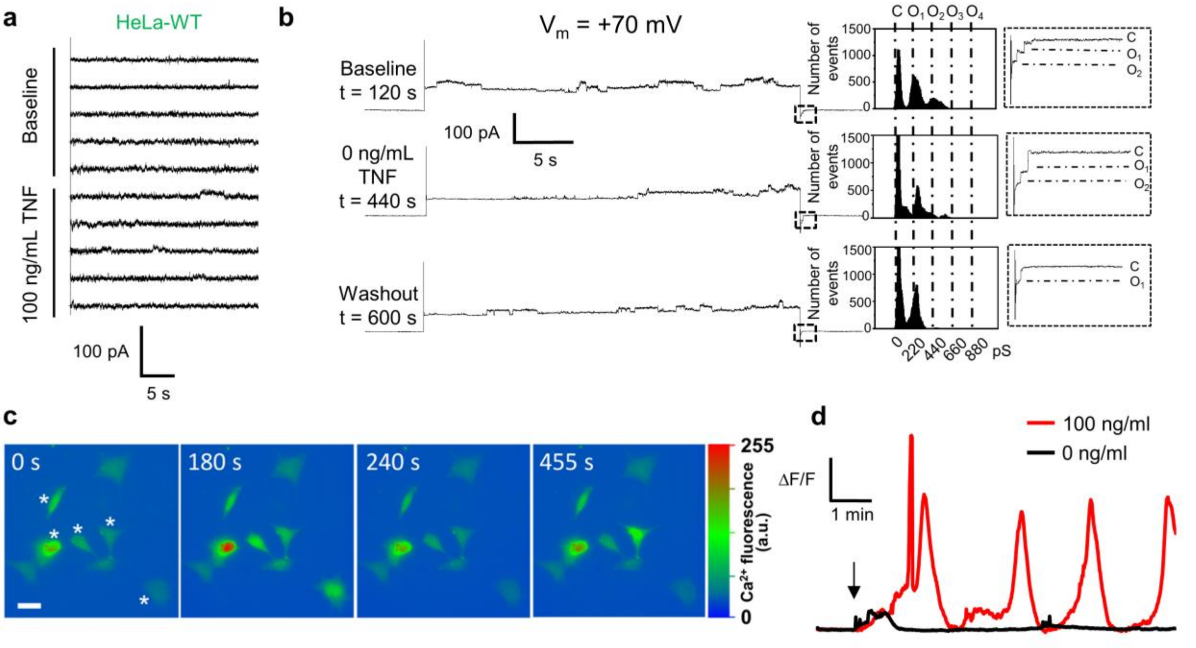

Supplemental Fig. 3: Control experiments and TNF induced Ca^{2+} responses. (a) Representative current traces obtained in HeLa-WT control cells, in baseline or stimulation with 100 ng/ml TNF. **(b)** Negative control experiment for the experiment shown in Fig. 3a performed here without TNF. **(c)** Representative images of HeLa-Cx43 cells depicting Ca^{2+} fluorescence changes upon exposure to 100 ng/ml TNF. Stars indicate cells displaying Ca^{2+} oscillations. Scale bar equals 20 µm; a.u. = arbitrary units. **(d)** Example trace demonstrating TNF-elicited Ca^{2+} oscillations (TNF addition at black arrow). In the absence of TNF, no Ca^{2+} oscillations were observed (black trace).

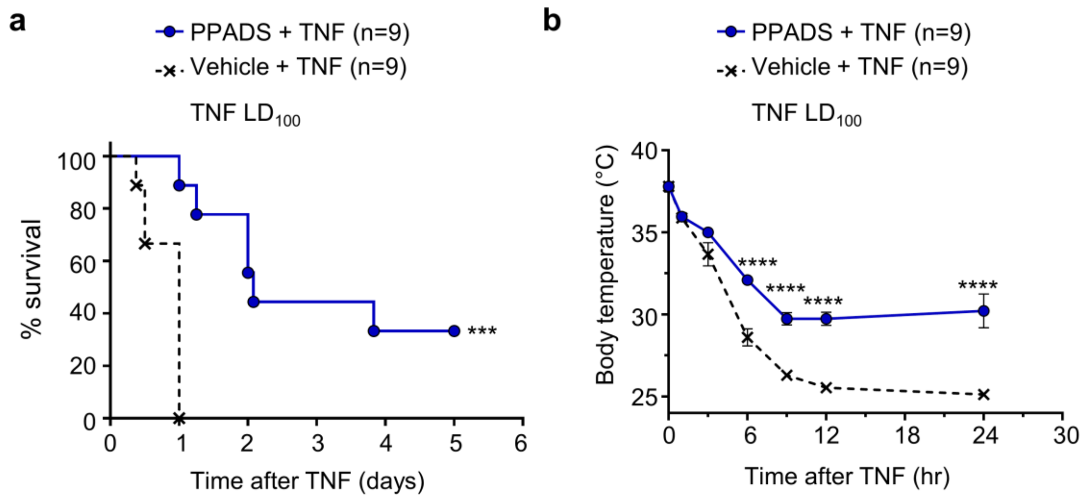

Supplemental Fig. 4: Interfering with the ATP sensing P2X- and P2Y-receptors by PPADS protects mice against TNF-induced mortality and hypothermia. (a-b) C57BL/6J mice were injected i.p. with vehicle (DPBS) or PPADS (50 mg/kg), 15 min before TNF injection (LD_{100}, i.v.). **(a)** Cumulative survival rates and **(b)** body temperatures presented as a function of time. Pool of 2 independent experiments; $^{****}p<0.0001$; $^{***}p=0.0002$. n = total number of mice per group. In (b), data are presented as mean ± SEM. Statistical tests used: (a) Mantel-Cox test; (b) 2-Way ANOVA with post-hoc Sidak's test.
